# Filth and Health

**Published:** 1859-08

**Authors:** 


### FILTH AND HEALTH.

Facts make no man wise. To be profited by them, we must not only see to it that they are whole facts, but we must have intelligence enough to be able to make a good use of them. At what an immense remove must they be from wisdom, who have neither facts nor common sense!

Some New Orleans savan or sapient, contended over his own signature, a very few years ago, that the prevalence of the epidemic was not fairly attributable to the then existing filthy condition of the streets; that if any thing, the more filthy parts of the city were the most lightly stricken.

Many otherwise sensible persons in passing along the street or public road, and seeing ruddy looking children clad in rags and begrimed with dirt, have jumped at the conclusion that playing in the dirt, was a means of health. It might just as well be argued that it is healthy to drink gin three times a day, because we find men at the age of four or five score, who from youth had kept up the habit, and lived in spite of it.

According to the most reliable accounts, more of the inhabitants of the Faroe Islands die between eighty and ninety years, than during any other decade of existence; and yet, travellers tell us, that around nearly every house, is a black feted sewer; the houses themselves being small and stifling, while the adjacent rill is defiled with the washing of clothes, and the eviscerations of fish. In all these cases, it would seem to be the ejaculation of common sense, how much longer these persons might have lived, in the observance of better habits of life. It would be about as wise as to contend that the extravagance of a spendthrift heir was a means of enriching, because he died rich, in spite of his extravagance. The tendency to argue in this manner has, in many directions, retarded the advance of wiser and better habits of life, Men may live long in spite of some pernicious habit, but without it, they would have lived longer. Incorrect reasonings in this regard have often ruined health, and shortened life; and will, in multitudes of instances, do it again.

The inhabitants of Iceland, and the distant Faroe Islands, lived long in the midst of the described filth, in part because the cold is so great there, that such filth was never heated to a degree which would make it unhealthful but for a very few days in the year; and this was at a season when they went on their annual fishing and hunting excursions, and consequently avoided exposure to hurtful exhalations, besides, hard necessity kept them from the excesses of civilized life.

